# The Brown Algae Pl.LSU/2 Group II Intron-Encoded Protein Has Functional Reverse Transcriptase and Maturase Activities

**DOI:** 10.1371/journal.pone.0058263

**Published:** 2013-03-11

**Authors:** Madeleine Zerbato, Nathalie Holic, Sophie Moniot-Frin, Dina Ingrao, Anne Galy, Javier Perea

**Affiliations:** 1 Inserm, U951 Evry, France; 2 University of Evry Val d’Essonne, UMR S_951, Evry, France; 3 Genethon, Evry, France; University of Poitiers, France

## Abstract

Group II introns are self-splicing mobile elements found in prokaryotes and eukaryotic organelles. These introns propagate by homing into precise genomic locations, following assembly of a ribonucleoprotein complex containing the intron-encoded protein (IEP) and the spliced intron RNA. Engineered group II introns are now commonly used tools for targeted genomic modifications in prokaryotes but not in eukaryotes. We speculate that the catalytic activation of currently known group II introns is limited in eukaryotic cells. The brown algae *Pylaiella littoralis* Pl.LSU/2 group II intron is uniquely capable of *in vitro* ribozyme activity at physiological level of magnesium but this intron remains poorly characterized. We purified and characterized recombinant Pl.LSU/2 IEP. Unlike most IEPs, Pl.LSU/2 IEP displayed a reverse transcriptase activity without intronic RNA. The Pl.LSU/2 intron could be engineered to splice accurately in *Saccharomyces cerevisiae* and splicing efficiency was increased by the maturase activity of the IEP. However, spliced transcripts were not expressed. Furthermore, intron splicing was not detected in human cells. While further tool development is needed, these data provide the first functional characterization of the PI.LSU/2 IEP and the first evidence that the Pl.LSU/2 group II intron splicing occurs *in vivo* in eukaryotes in an IEP-dependent manner.

## Introduction

Prokaryotic and eukaryotic organelle introns are mobile elements able to integrate specifically in the exon junction of an intronless genome [1]–[3]. This property, called “homing”, contributes to the spreading of introns and has been used for precise *in vivo* genome engineering [4]–[13]. Two general homing mechanisms have been described depending on the type of intron: group I introns encode a very specific nuclease (meganuclease) to produce a double-strand break (DSB) in the intronless genome at the junction of exons. The DSB is then repaired by homologous recombination (HR) with a template DNA coming from the “invader” genome [14]. Group II introns are ribozymes that self-splice from precursor RNA yielding excised intron lariat RNAs. The lariat splicing intermediate recognizes the DNA junction between the exons of the intronless genome and integrates the genome forming a DNA-RNA hybrid. After reverse transcription, the template intronic RNA is degraded and the gap is repaired by a DNA polymerase. In spite of very divergent mechanisms used for homing, both group I or group II introns rely on the expression of a protein coded by the intron itself (IEP, Intron-Encoded Protein) for the homing process [2]. These IEPs often carry different activities: maturase (to help the proper splicing of the intron) [15]–[22], double strand endonuclease (in group I introns) [17], [18], [20], [22], single strand endonuclease and reverse transcriptase (in group II introns) [23]–[28]. Homing always results in the incorporation of a copy of the intron into the intronless genome.

The ability of group I and group II introns to recognize and to integrate into a specific genomic site has been exploited to generate various knock-out or knock-in models in mammalian cells [4], [12], [13], [29], [30], plants [31]–[33], and bacteria [5], [34]–[38]. Specific genomic targeting is obtained by changing the native target recognition sequences using rational engineering or directed molecular evolution [13], [39]. At present, group II intron-derived genomic targeting strategies are only used in bacteria. However, using group II introns in mammalian cells could represent some advantages over the currently existing technologies. In mammalian cells, meganucleases and strategies based on FokI restriction endonuclease coupled to engineered Zinc fingers [32] or Transcription Activator-Like Effectors [40], [41] are used for specific genomic insertion. Both of these approaches utilize DSB repair processes which occur mainly by non-homologous end joining (NHEJ) or by HR in the presence of a DNA template [42]–[44]. These approaches are limited by safety concerns and by low efficiency in some cell types. The radically different homing mechanism of group II introns could be an alternative to DSB mediated gene engineering.

There are only a few examples of the use of group II introns in eukaryotes. An initial proof of concept in mammalian cells has been reported by Guo *et al.* [4] using the *Lactococcus lactis* Ll.LtrB intron to target two genes carried into plasmids in HEK 293 human cells. These initial experiments showed homing into the plasmid as detected by PCR with specific oligonucleotides but efficacy was not measured. The splicing of the Ll.LtrB intron inside the HEK 293 cells was also not demonstrated since the authors introduced directly the purified LtrA-lariat ribonucleoparticles into the cell to obtain homing. However, it is known that the Ll.LtrB intron can acquire its correct tertiary structure in bacteria with the help of LtrA as used in commercial gene knock-out systems [5], [45]–[47].

Several elements may contribute to the limitations in the use of group II introns in eukaryotes. Ribozyme activity of group II introns depends on the correct folding of RNA into a specific tertiary structure [48]. This activity is necessary for both the intron splicing and its insertion into target DNA. Most of group II introns are able to self-splice *in vitro* in the absence of proteins but have to be “chaperoned” by proteins *in vivo* [48], [49]. These proteins may not function optimally in eukaryotic cells. Self-splicing of group II introns depends on the correct tertiary structure folding of the intronic RNA, and Mg^2+^ cations contribute to this folding by stabilizing the RNA tertiary structure [48], [50]. Mg^2+^ is also required for catalysis of group II introns [51] and its concentration is critical for proper self-splicing *in vitro*. It is therefore possible that most group II intron cannot function optimally in eukaryotic cells where the free Mg^2+^ concentration is estimated to be around 1–2 mM [52]. For example, the optimal *in vitro* reaction conditions for the well-known bacterial *Lactococcus lactis* Ll.LtrB intron IEP-assisted RNA splicing use 5 mM Mg^2+^ and 10 mM of Mg^2+^ are required for an optimal reverse splicing reaction of RNPs into DNA target site [53]. The importance of Mg^2+^ concentration is emphasized by a recent study showing that Ll.LtrB RNP particles were able to insert efficiently into a plasmid target in eukaryotic nuclei by providing MgCl_2_ during the microinjection of RNPs in *Xenopus laevis* oocytes, and *Drosophilia melanogaster* and zebrafish (*Danio renio*) embryos [29]. In this work, we have chosen to study the Pl.LSU/2 intron from the mitochondrial large subunit rRNA gene of the brown algae *Pylaiella littoralis* because of its ability to self-splice *in vitro* at unusually low Mg^2+^ concentrations (0.1 mM) [54]. We reasoned that this characteristic could make the Pl.LSU/2 intron a good candidate to be used as a tool for genome manipulation in eukaryotic cells. The Pl.LSU/2 intron structure presents a highly canonical secondary structure model consisting in six helical domains (I to VI) radiating from a central wheel [55], [56]. Although the Pl.LSU/2 intron tertiary structure has been characterized [57]–[59], there is no report on the homing property of this intron nor on its potential biochemical activities *in vivo*. Moreover, the putative intron-encoded protein of PI.LSU/2 has not been described. The fact that the Pl.LSU/2 intron sequence presents in its domain IV an open reading frame containing putative domains of most group II intron IEPs [56] suggests that at least one of the Pl.LSU/2 IEP putative activities has been preserved by evolution.

We have therefore characterized the biochemical activities of the Pl.LSU/2 IEP as well as the Pl.LSU/2 intron activity *in vivo* in eukaryotic cells in order to evaluate the possibility of using this intron in genome engineering. Here, we show that active recombinant Pl.LSU/2 IEP can be produced and purified in *Escherichia coli*. The purified IEP shows a reverse transcriptase activity *in vitro* both alone or when expressed together with the intronic RNA. The Pl.LSU/2 intron is able to splice properly in yeast cells helped by the maturase activity of its IEP.

## Materials and Methods

### Strains and Human Cell Lines

Pl.LSU/2 intron and Pl.LSU/2 intron-encoded protein were expressed in *E. coli* strain Rosetta-gami B(DE3) F^–^ *ompT hsdS*_B_ (r_B_^–^ m_B_^–^) *gal dcm lacY1 ahpC* (DE3) *gor522*::Tn*10 trxB* pRARE (Cam^R^, Kan^R^, Tet^R^) (EMD4Biosciences, Novagen). This strain was grown in LB (Luria-Bertoni) medium with 50 µg/ml of carbenicilin and/or 25 µg/ml of chloramphenicol. *In vivo* splicing of Pl.LSU/2 intron was evaluated in *S. cerevisiae* BY4742 *MAT*α *his3*Δ*1 leu2*Δ*0 lys2*Δ*0 ura3*Δ*0* (S288C) [60]. This strain was grown in YPD (Yeast extract Peptone Dextrose) and/or in SD (Synthetic Dextose) medium containing dropout supplement mix (according to Molecular Cloning, A Laboratory Manual, by Sambrook J. & Russell D.). HEK 293T and HCT 116 human cells (obtained from ATCC (CRL-11268 and CCL-247 respectively, American Type Culture Collection, Manassas, VA, USA) were grown in DMEM supplemented with 10% fetal bovine serum (FBS) and 1% penicillin/streptomycin (PS) (Life technologies, Invitrogen). Cells were passaged with TrypLE express 1X (Life technologies, Invitrogen).

### Plasmids and Oligonucleotides

Plasmids and oligonucleotides used in this study are listed in Supplemental information (Table S1 A and Table S1 B, respectively).

### Structure Analysis

Pl.LSU/2 intron secondary structure has already been described [55], [56]. The secondary structure of the intron RNA domain IV was predicted with the sFold 2.2 software (http://sfold.wadsworth.org) [61], [62] (Fig. S2).

### Expression of the Pl.LSU/2 Intron-encoded Protein (IEP) in *E. coli*

*E. coli* strain Rosetta-gami B (DE3) was transformed with the appropriate expression plasmid and single colonies were inoculated into 4 ml of LB containing appropriate antibiotics. Precultures were shaken at 170 rpm at 32°C overnight, inoculated into 100 ml of LB medium without antibiotics and grown at 32°C for 3–6 hr, until OD_600 nm_ reached 0.4–0.8. Induction was started by addition of IPTG (1 mM final), and the incubation was continued for 3 hr at 30°C. Cells were then collected by centrifugation (1,900 *g* for 10 min at 4°C), and washed once with 20 ml of 150 mM NaCl. The washed cell pellet was stored at −80°C overnight.

### Purification of the Pl.LSU/2 IEP and RNP Particles by Sucrose Cushion Centrifugation

The IEP, tagged in N-terminus with an histidine stretch (6xHis) and a V5 epitope (GKPIPNPLLGLDST) was purified by sucrose cushion centrifugation, as described [23]. The washed cell pellet was resuspended in 4 ml of ice-cold buffer A (50 mM Tris-HCl at pH 7.4, 1 mM EDTA, 1 mM DTT, 10% (v/v) glycerol), and lysozyme was added to a final concentration of 4 mg/ml. After 45 min of incubation on ice, cells were lysed by three cycles of freeze/thawing between −70°C and 37°C, followed by addition of 2.5 volumes of HKCTD buffer (25 mM Tris-HCl at pH 7.4, 500 mM KCl, 50 mM CaCl_2_, 5 mM DTT). Lysate was then centrifuged (14,000 *g* for 15 min at 4°C), and supernatant was layered over 5 ml of 1.85 M sucrose containing HKCTD and centrifuged in a Beckman Type 70 Ti rotor (50,000 *g* for 17 h at 4°C). The resulting pellet was gently washed with 1 ml of ice-cold Milli-Q water and then dissolved in 25 µl of ice cold 10 mM Tris-HCl at pH 8.0, and 1 mM DTT. IEP mtDD- and RNP particles containing IEP WT or IEP mtDD- were purified according to the same procedure. Purified proteins and RNPs preparations were stored at −80°C. The yield of RNP particles was 25–90 OD_260 nm_ units per 100 ml of culture, with 1 OD_260 nm_ unit of RNP containing 0.84±0.12 µg of IEP. The yield of purified proteins was 55–150 µg per 100 ml of culture. Quantification of proteins was performed by using Bio-Rad DC Protein Assay Kit 1 (BioRad Laboratories) according to the manufacturer’s instructions. All protein fractions were analyzed by Coomassie-blue staining of SDS-PAGE and western blotting. Nucleic acids in IEP and RNPs (WT and mtDD-) purified fractions were quantified by spectrophotometry (NanoDrop 8000) and analyzed by electrophoresis on agarose gel.

### RNA Extraction

Total cellular RNA from yeast cells was extracted from 5 ml of mid-log phase cultures in SD minimal medium (minimal SD base or minimal SD base Gal/Raf, Clontech). After centrifugation (3,800 *g* for 5 min at 4°C), the cell pellet was washed with 1 ml of Milli-Q water and resuspended in 2 ml of buffer Y1 (1 M sorbitol, 0.1 M EDTA) supplemented with 0.1% β-mercaptoethanol and 300 U of lyticase (from *Arthrobacter luteus*, >2,000 units/mg protein, Sigma). After 30 min of incubation at 30°C on a rotary shaker, total cellular RNA was extracted from the resulting spheroplasts using the RNeasy Mini Kit (Qiagen) according to the manufacturer’s instructions. Total cellular RNA from human cells was also isolated with RNeasy Mini Kit (Qiagen). In both cases, a DNase I treatment (RNase-free DNase set, Qiagen) was performed on-column during RNA purification according to the manufacturer’s instructions.

### Protein Extraction

To obtain yeast total proteins, 20 ml of culture were centrifuged (3,800 *g* for 5 min at 4°C). The cell pellet was washed with 5 ml of Milli-Q water, resuspended in CelLytic Y reagent (2.5 ml/g cell pellet, Sigma-Aldrich) supplemented with 7 mM DTT and incubated at 25°C for 20 min on a rotary shaker. The resulting lysate was centrifuged at 18,000 *g* for 10 min at 4°C. Proteins were then precipitated with 2 volumes of acetone for 1 hr at 4°C and recovered by centrifugation at 18,000 *g* for 15 min at 4°C. Protein pellet was then washed with ethanol and resuspended in 0.5 volume of 50 mM Tris-HCl at pH 8.5, 8 M Urea, and 10 mM DTT.

Yeast nuclear proteins were extracted from 200 ml of culture after centrifugation (4,000 *g* for 5 min at 4°C). Cell pellets were washed first with 25 ml of Milli-Q water and then with 3 ml of spheroplasting buffer (1 M Sorbitol, 50 mM K_2_HPO_4_ at pH 6.5, 0.018% β-mercaptoethanol). Cells were resuspended in 3 ml of spheroplasting buffer containing 500 U of lyticase and incubated 30 min at 30°C on a rotary shaker. The spheroplasts were collected by centrifugation (4,500 *g* for 5 min at 4°C), washed with 3 ml of ice-cold spheroplasting buffer, resuspended in 8 ml of ice-cold buffer L (18% Ficoll 400, 20 mM K_2_HPO_4_ at pH 6.8, 1 mM MgCl_2_, 0.5 mM EDTA, 2 mM PMSF, 1 µg/ml aprotinin) and lysed on ice by 20 strokes of a dounce homogeneizer. The resulting lysate was then centrifuged (3,500 *g* for 10 min at 4°C). Supernatant was then centrifuged in a Beckman SW 55 Ti rotor (58,000 *g* for 35 min at 4°C) and pelleted nuclei were resuspended in 200 µl of ice-cold buffer NP (0.34 M sucrose, 20 mM Tris-HCl at pH 7.4, 50 mM KCl, 5 mM MgCl_2_, 2 mM PMSF, 1 mg/ml aprotinin). Nuclear protein extracts were stored at −80°C.

Human total protein extracts were prepared following washing of cells and lysis of the pellets in buffer containing 50 mM Tris-HCl pH7.5, 200 mM NaCl, 1 mM EDTA, 1 mM PMSF, 1% Triton X-100, 0.1% SDS, 0.5% sodium deoxycholate, and 10% glycerol supplemented with protease inhibitors cocktail.

Protein concentrations were determined with the Bio-Rad DC Protein Assay kit 1.

### Western Blot Analyses

Proteins were resolved on a denaturing 10% polyacrylamide-SDS gel (Criterion XT Bis-Tris gels, Biorad), transferred onto a nitrocellulose membrane (Hybond ECL, Amersham) and probed with the appropriate antibodies.

Immunoblots of the 6xHis/V5-tagged Pl.LSU/2 IEP expressed in *E. coli* were probed with mouse anti-V5 antibody (1∶5,000 dilution, Life technologies, Invitrogen) followed by IRDye 680-conjugated goat anti-mouse antibody (1∶8,000 dilution, LI-COR Biosciences) and immunoreactive bands were detected with the Odyssey infrared scanner (Li-Cor).

Yeast total proteins (30 µg/lane) were immunoblotted with mouse anti-HA antibody (1∶200 dilution, Santa Cruz Biotechnologies) and rat anti-Tub1p antibody (1∶5,000 dilution, Abcam) to detect respectively the expressed IEP and Tubulin 1 protein (Tub1p), then with Horseradish peroxidase (HRP)-conjugated goat anti-mouse antibody (1∶25,000 dilution, Jackson ImmunoResearch) and HRP-conjugated rabbit anti-rat antibody (1∶1,000 dilution, Dako). Bands were revealed by chemiluminescence (Supersignal West Dura Extended Duration Substrate, Thermoscientific).

Yeast nuclear proteins (80 µg/lane) and human total proteins (30 µg/lane) were probed with an HRP-conjugated mouse anti-c-Myc antibody (1∶3,000 dilution, Life technologies, Invitrogen) to detect the myc-tagged NLS-IEP. For yeast nuclear proteins, a mouse anti-TATA binding protein (TBP) antibody (final concentration of 2 µg/ml, Abcam) was added and HRP-conjugated goat anti-mouse antibody (1∶20,000 dilution) was used as secondary antibody. Bands were revealed by chemiluminescence.

### RT Assays

RT activity was assayed for 45 min at 37°C in 14 µl of reaction medium containing 10 mM KCl, 10 mM MgCl_2_, 50 mM Tris-HCl at pH 8.0, 5 mM DTT, 0.05% NP40, 1 µg of poly(rA)-oligo(dT)_12–18_ (Amersham), 10 µCi of [α-^32^P]dTTP (3,000 Ci/mmole, Perkin-Elmer) and 1 µg of RNase A (Sigma Aldrich), as described [23]. Reactions were started by the addition of either IEP or RNPs preparations and stopped by addition of EDTA (50 mM final). Radioactive products were spotted on a DE81 filter (Whatman) which was washed twice in 2X SSC. After an overnight exposure on a phosphor screen (Molecular Dynamics PhosphorImager System; GE Healthcare Bio-Sciences), radioactive spots were detected by the Storm system (GE-Healthcare Bio-Sciences) and data were analyzed with ImageQuant software (GE Healthcare Life Sciences).

### Reverse Transcription

Reverse transcription of yeast or human cellular RNA was carried out in 20 µl of reaction medium using the Verso cDNA Kit (Thermo Scientific). One microgram of total RNA was incubated for 5 min at 70°C and then mixed with 1X reverse transcription buffer, 0.5 mM of each dNTPs, 2 µM of RM-R oligonucleotide (yeast RNA; Table S1 B) or 300 ng of random hexamers and 125 ng of anchored oligo dT (human RNA), 1 µl of RT Enhancer enzyme, and 1 µl of Verso reverse transcriptase. After incubation at 42°C for 1 hr, reaction was stopped by heating 2 min at 95°C. A control reaction without Verso reverse transcriptase was performed to ensure the absence of DNA contamination in the RNA samples (minus-Verso RT samples).

### PCR

Two µl of yeast cDNA were used as a template for PCR in 50 µl of reaction medium containing 1 unit of Phusion high-fidelity DNA polymerase (Thermoscientific), 62.5 µM of each dNTPs, PCR buffer, and 0.2 µM of each p1 and p2 or p3 and p4 primers. The amplification consisted of 30 cycles at 98°C for 30 sec, 70°C for 30 sec and 72°C for 30 sec. PCR products were analyzed on 12% polyacrylamide gels.

Two µl of human cDNA were used as a template for PCR in 40 µl of reaction medium containing 0.8 unit of Phusion high-fidelity DNA polymerase, 200 µM of each dNTPs, PCR buffer, and 0.25 µM of each p5 and p6 primers. The amplification consisted of 30 cycles at 98°C for 10 sec, 58°C for 20 sec and 72°C for 1 min 30 sec. PCR products were analyzed on 1.2% agarose gels.

### Quantitative PCR

The qPCR consisted of a SYBR Green-based detection of cDNA with specific primers hybridizing E2 and the E2–E3 junction or primers hybridizing E2 and the E2-Intron junction. Amplification reactions (25 µl) contained 5 µl of a 1/10 dilution of cDNA and 12.5 µl of qPCR buffer (Power SYBR Green PCR master mix, Applied Biosystems), 0.3 µM of each primers and consisted of 40 cycles at 95°C for 15 sec then at 65°C for 1 min on a 7900 HT Real-Time PCR System (Applied Biosystems). To ensure the absence of non-specific amplifications, a dissociation step was added consisting in a final cycle at 95°C (15 sec) then 65°C (15 sec) and finally 95°C (15 sec). To quantify cDNA copy number obtained respectively from spliced or precursor mRNA, standard amplification curves were made by serial dilutions of appropriate linearized plasmids. All PCR measures were performed at least in duplicate. All qPCR experiments include samples from mock vector-transformed cells and minus-Verso RT samples as negative controls.

Data were edited using the Sequence Detection Systems 2.3 software (Applied Biosystems) and interpreted in the linear portion of the standard curve. The following test acceptability criteria were used: linear regression coefficient of the standard curve >0.98; less than 0.5 CT variation for duplicate samples; CT comprised between 35 and 40 for H_2_O; mock vector-transformed cells and minus-Verso RT samples.

### Generation and Titration of Lentiviral Vectors

Recombinant lentiviral vectors (LVs) using the pRRL backbone plasmid [63] (Table S1 A) were constructed to express the indicated transgenes under the control of the human phosphoglycerate kinase (PGK) promoter. VSV-G-pseudotyped particles were produced by quadritransfection of HEK 293 T cells (Table S1 A) as previously described [63], [64]. Harvested virus particles were concentrated by ultracentrifugation and titered in infectious genome particles (IG) as previously described [64].

### Lentiviral Vector Transduction of Human Cell Lines

HCT 116 cells were seeded at 10^5^ cells per well in 12-well plates 16–24 hrs prior transduction. Cells were transduced with LVs using concentrations ranging from 10^6^ to 10^8^ IG/ml in the presence of 6 µg/ml of polybrene. Transduction medium was replaced with fresh medium 6 hrs after transduction. A second hit of transduction was performed the following morning in the same conditions using LVs encoding proteins. Cells were removed and analyzed 48 hrs later. For LVs stably expressing different forms of Pl.LSU/2 intron, cells were expanded for about 10 days. Vector copy numbers per cell were calculated by quantitative PCR on cell lines genomic DNA as previously described [64] (0.9 for intron-ΔDIV; 0.3 for intron-DIVa; 4.7 for intron-DIVab, and 1.2 for full length intron).

## Results

### Pl.LSU/2 IEP Contained in RNP Particles Presents an RT Activity *in vitro*

**Figure 1:**
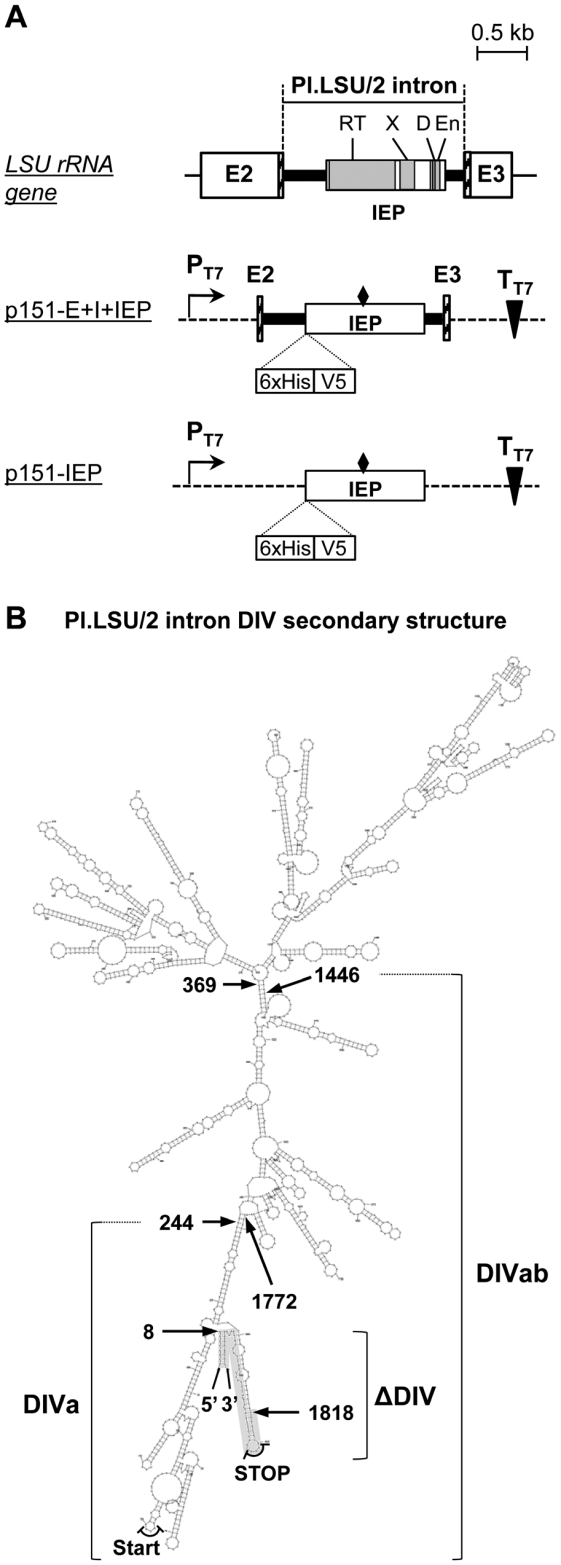
Schematic representation of plasmids and Pl.LSU/2 intron domain IV used. (*A*) Gray rectangles: Predicted protein domains shared by other group II intron ORFs; E2 and E3: exons flanking the Pl.LSU/2 group II intron [56]; bold line: Pl.LSU/2 group II intron; broken line: vector sequences; PT7: Specific promoter of the T7 bacteriophage RNA polymerase; TT7: T7 bacteriophage RNA polymerase transcription terminator; 6xHis: histidine tag; V5: V5 epitope; hatched rectangles: 50 last nt of E2 and 71 first nt of E3; diamond: position in which the RT catalytic motif YADD is mutated in YAAA on negative control plasmids (p151-E+I+IEPmtDD- and p151-IEPmtDD-). (*B*) Predicted RNA secondary structure of Pl.LSU/2 intron domain IV (DIV). Start and Stop codons of the IEP ORF are indicated. DIVa: section conserved in the intron-DIVa form (deletion from 244 to 1772 nt of DIV). DIVab: section conserved in the intron-DIVab form (deletion from 369 to 1446 nt of DIV). ΔDIV : section conserved in the intron-ΔDIV form (section highlighted in gray); the section from 8 to 1818 nt of DIV is replaced by the sequence CCTAGGATCT [54]. The detailed putative secondary structure is available in Fig. S2.

The *Pylaiella littoralis* Pl.LSU/2 group II intron is located in the mitochondrial gene encoding the large ribosomal RNA (Fig. 1A; LSU rRNA gene). This intron contains in its domain IV an open-reading frame presenting the predicted conserved domains of group II intron-encoded proteins which are the reverse transcriptase (RT), DNA-binding domain (D), maturase (X) and endonuclease (En) [15], [23], [25], [28], [53] (Fig. 1A). Subsequently, recombinant Pl.LSU/2 intron-encoded protein (IEP) was expressed and purified in order to measure its predicted biochemical activities.

#### Expression of IEP in RNP particles

Some group II intron-encoded proteins are known to be fully active either alone [53], [65] or when intron RNA is copurified with the expressed IEP [23]. To express the Pl.LSU/2 IEP in RNP particles, we designed the plasmid p151-E+I+IEP for the induction of both the Pl.LSU/2 intron and IEP expression in *E. coli* (Fig. 1A). This plasmid contains the Pl.LSU/2 intron and its flanking exons (50 last nucleotides of exon 2 and 71 first nucleotides of exon 3) [54] cloned downstream of the phage T7 promoter in the expression vector pET151/D-TOPO (Table S1 A). The IEP ORF is fused to a 6xHis and a V5 epitope tags in its N-terminus used to the detection of the protein by western blot. This vector could potentially allow the expression of both the intron RNA and the wild-type IEP ORF (IEP WT) with its own Shine-Dalgarno sequence for translation. We also constructed a derivative negative control plasmid (p151-E+I+IEPmtDD-) in which the catalytic YADD motif of the IEP RT domain is mutated to YAAA (Fig. 1A, position indicated by a diamond; Table S1 A) [66]. This plasmid is expected to express a mutant RT-defective IEP (IEP mtDD-).

**Figure 2:**
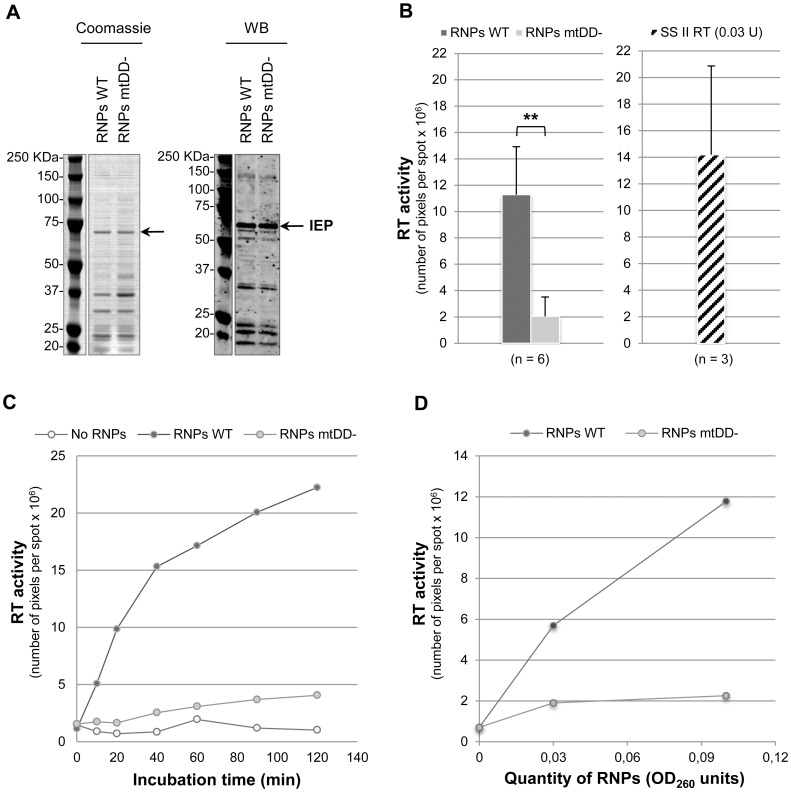
RT activity of Pl.LSU/2 IEP in RNP particles purified from *E. coli*. (*A*) SDS-PAGE analysis of IEP-containing RNPs preparations by Coomassie-blue staining (Coomassie) and western blot (WB). Quantities of RNPs used were 9 OD_260 nm_ units for RNPs with IEP WT (RNP WT) and 18 OD_260 nm_ units for RNPs with IEP mtDD- (RNP mtDD-). A monoclonal mouse anti-V5 antibody was used to detect the IEP by western blot. The IEP is indicated by the black arrow. Numbers at *left* indicate molecular mass markers in kilodaltons (KDa). (*B*) RT assays with 0.1 OD_260 nm_ units of RNPs. Dark gray bar: RNPs containing IEP WT; Light gray bar: RNPs containing IEP mtDD-; Hatched bar: control SuperScript® II reverse transcriptase (SS II RT). Data represent the number of pixels per spot indicating the [α-^32^P]dTTP incorporation for each reaction. Data are the means of at least three independent experiments with the standard deviation indicated by thin lines. Data were subjected to a *t*-test using a unilateral pair-wise comparison procedure. A highly significant difference is indicated by asterisks (*p*<6 x 10^−4^). (*C*) Time course of RT reaction. RT activity of RNPs containing IEP (RNPs WT, dark gray dots) was assayed with 0.1 OD_260 nm_ units of RNPs. Reactions were performed using various incubation times. Experiments were also performed without RNP particles (No RNPs, light gray dots) or with RNP particles containing the mutant Pl.LSU/2 IEP mt DD- (RNPs mtDD-, gray dots). (*D*) Dose-dependent effect on RT activity. RT activity of RNPs containing IEP (RNPs WT, dark gray dots) or mutant IEP (RNPs mtDD-, light gray dots) was assayed with different quantities of RNPs for 45 min.

The expression plasmids p151-E+I+IEP and p151-E+I+IEPmtDD- were transformed in *E. coli* Rosetta-gami B (DE3). After induction, soluble protein fractions were used to purify RNP particles by sucrose cushion centrifugation [23]. Bands at the expected size of IEP WT and mtDD- are observed in the Coomassie-stained SDS-PAGE gel (Fig. 2A; Coomassie, 69 kDa, black arrow). Other bands are also detected by Coomassie staining and correspond to *E. coli* contaminant proteins and/or IEP (WT and mtDD-) degradation products. The western blot analysis confirms the presence of IEP WT and mtDD- at the expected size (Fig. 2A; WB) and shows the presence of some degradation products. A non-specific band is also detected by western blot at 150-kDa in RNPs WT and mtDD- preparations (Fig. 2A; WB). The analysis of these RNPs fractions by agarose gel electrophoresis shows the presence of ribosomal RNAs (Fig. S1). RNP particles containing either IEP WT or IEP mtDD- can thus be partially purified using sucrose cushion centrifugation.

#### RT activity

The open reading frame of the intron Pl.LSU/2 contains a conserved RT domain. The RT activity of Pl.LSU/2 IEP (WT and mtDD-) in RNP particle preparations was assayed with the artificial template-primer substrate poly(rA)-oligo(dT)_12–18_. We show that RNP particles from cells expressing p151-E+I+IEP and purified by sucrose cushion centrifugation have an RT activity (Fig. 2B; RNP WT). Quantitatively, the RT activity of 0.1 OD_260 nm_ units of RNP particles is similar to that of 0.03 units of the commercial SuperScript® II reverse transcriptase (SSII RT; Life technologies, Invitrogen). The time course of RT reactions shows that the RT activity of WT IEP-containing RNPs increases over incubation time (Fig. 2C) and is positively correlated with the amount of RNP particles (Fig. 2D). As expected, the RT activity of the Pl.LSU/2 IEP is abolished by point mutations in the conserved YADD motif (Fig. 2B, 2C and 2D; RNP mtDD- conditions) and is similar to that of the background condition (Fig. 2C; No protein). The 6xHis tag was also used to purify RNP particles by immobilized metal-ion affinity chromatography (IMAC) on a Ni^2+^-charged column but no RT activity was ever detected in those RNP particle preparations (data not shown), suggesting that this purification process destabilizes the complex. These results indicate that the Pl.LSU/2 IEP in RNPs particles can be expressed in *E. coli* and purified by sucrose centrifugation thereby preserving its RT activity and thus its active conformation.

### Pl.LSU/2 IEP is Active *in vitro* without the Help of the Intron RNA

#### Isolated Pl.LSU/2 IEP has *in vitro* RT activity

**Figure 3:**
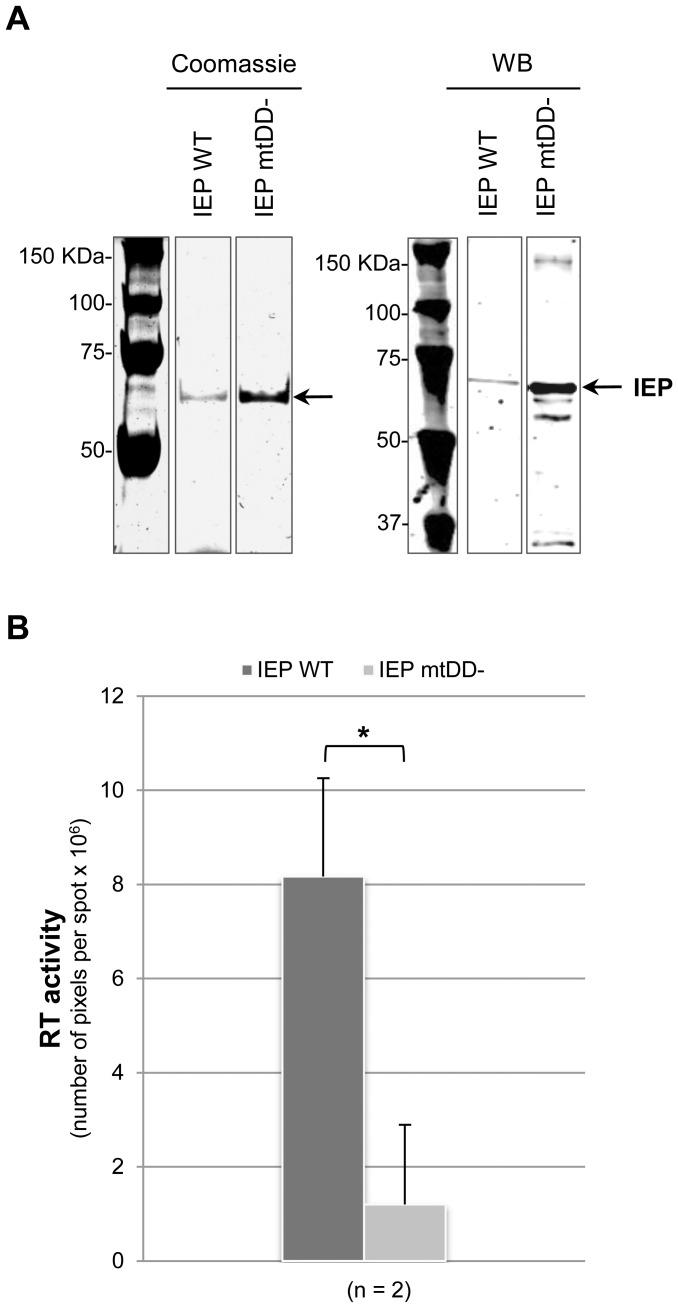
RT activity of Pl.LSU/2 IEP purified from *E. coli*. (*A*) Analysis of IEP purification by SDS-PAGE with Coomassie-blue staining (Coomassie) and western blot (WB). Volumes loaded contain 5–10 µg of purified protein fraction. A monoclonal mouse anti-V5 antibody was used to detect the IEP by western blot. The IEP is indicated by the black arrow. Numbers at *left* indicate molecular mass markers in kilodaltons (KDa). (*B*) RT assays with 100 ng of IEP. Dark gray bar: IEP WT; Light gray bar: IEP mtDD-. Data are the mean of two independent experiments with the standard deviation indicated by thin lines. Data were subjected to a *t*-test using a unilateral pair-wise comparison procedure. A significant difference is indicated by asterisk (*p*<0.036).

To analyze the intrinsic property of the Pl.LSU/2 IEP, we expressed the IEP in *E. coli* without coexpressing the intron RNA. We used the plasmid p151-IEP (Fig. 1A) which places the IEP ORF fused to the 6xHis and V5 epitope tags immediately downstream of the phage T7 promoter and Shine-Dalgarno sequence of the vector. This plasmid version was also constructed with a mutation of the YADD motif (Fig. 1A; indicated by a diamond). Both WT and mutant proteins were expressed in *E. coli* and then purified by sucrose cushion centrifugation. SDS-PAGE shows that the WT and the RT-defective IEPs (mtDD-) are successfully purified from *E. coli* lysates (Fig. 3A; Coomassie). The identity of the purified proteins is verified by western blot (Fig. 3A; WB) and by mass spectrometry (data not shown). It is worth noting that these purified protein fractions contain substantial amount of nucleic acids, in particular ribosomal RNAs (Fig. S1).

RT assays with Pl.LSU/2 IEP purified by sucrose cushion centrifugation show that the protein displays an intrinsic *in vitro* RT activity (Fig. 3B; IEP WT). This activity is abolished by mutations in the RT domain as expected (Fig. 3B; IEP mtDD-). The positive control SuperScript® II Reverse transcriptase (0.03 U) shows a number of pixels per spot of 18.1 x 10^6^±5.78 (not shown). It is noteworthy that no RT activity is detected following IMAC purification of IEP (WT and mtDD-, data not shown), which is consistent with previous findings obtained with RNPs. These data suggest that purification in sucrose cushion preserves the activity of the IEP and thereby its correct folding. It also demonstrates that recombinant IEP has intrinsic RT activity and does not necessarily require the help of intron RNA to become active.

### Splicing Assay of Pl.LSU/2 Intron in *S. cerevisiae*

*In vitro* self-splicing of Pl.LSU/2 has been demonstrated [54] using a deleted form of the intron in the domain IV (See Fig. 1B; intron-ΔDIV). However, most group II introns need the maturase activity of their IEP to achieve *in vivo* splicing. In *Lactococcus lactis* Ll.LtrB group II intron, the LtrA intron-encoded protein interacts with a substructure located in the beginning of the domain IV, and other contacts are made with the conserved core regions of the intron [27], [67]. In order to evaluate the influence of the IEP on the splicing of the Pl.LSU/2 intron in yeast, we included a part of the domain IV in our construction. This domain corresponds to DIVab indicated in the secondary structure of the domain IV (1870 nucleotides) predicted by the S-fold software (See Fig. 1B; Fig. S2). This DIVab domain retains putatively most of the predicted secondary structure of domain IV (S-fold prediction, not shown).

**Figure 4:**
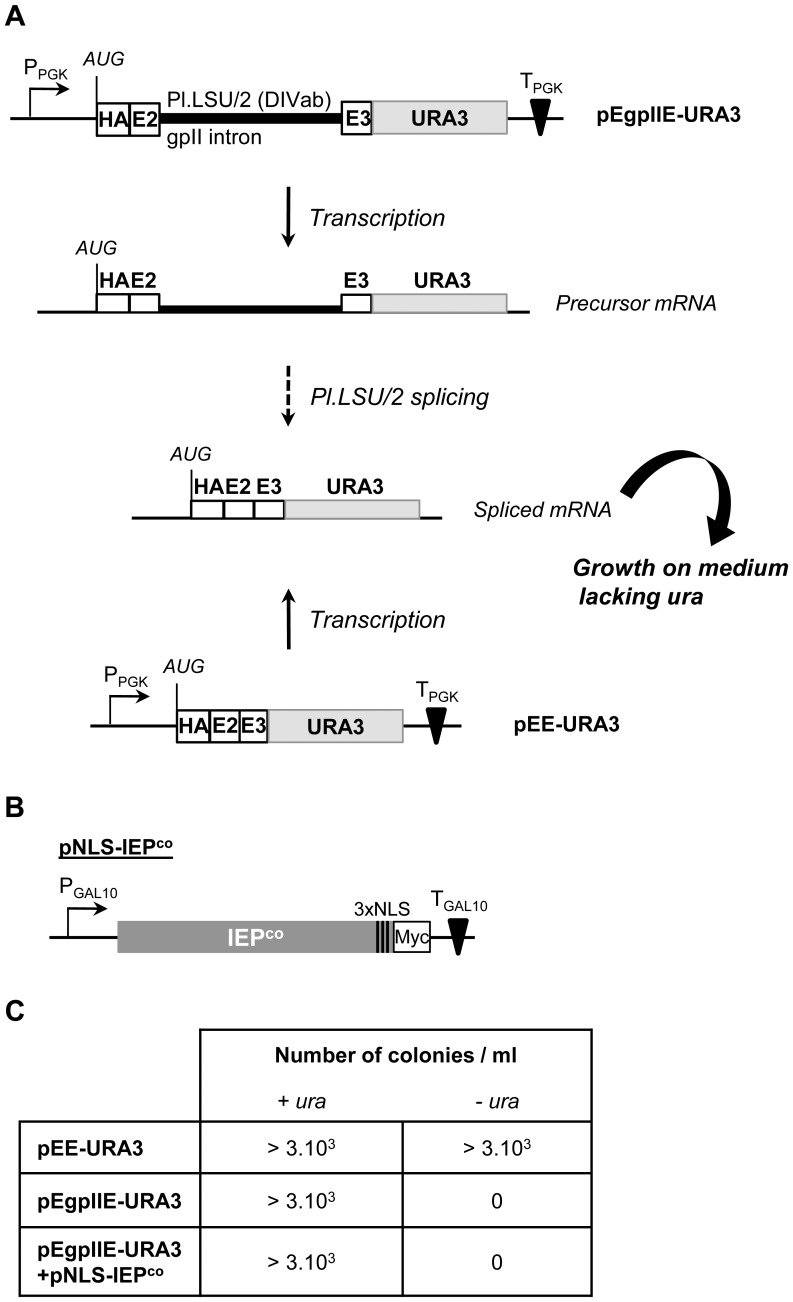
*In vivo* splicing assay of Pl.LSU/2 intron in *Saccharomyces cerevisiae*. (*A*) Schematic representation of the group II intron splicing reporter assay. PPGK: Phosphoglycerate kinase gene promoter; TPGK: Phosphoglycerate kinase gene transcription terminator; E2 and E3∶50 last nt of exon 2 and 71 first nt of exon 3; bold line: Pl.LSU/2 intron-DIVab; light gray rectangle: URA3 ORF; HA: HA epitope; AUG: Translation start codon. See text for detailed description of the assay. (*B*) Schematic representation of the NLS-IEP^co^ expressing plasmid. PGAL10: UDP-Galactose epimerase gene promoter; TGAL10: UDP-Galactose epimerase gene transcription terminator; dark gray rectangle: human codon-optimized Pl.LSU/2 IEP ORF (IEP^co^); 3xNLS: nuclear localization signals; Myc: stretch of 3 c-Myc epitopes. (*C*) Numbers of yeast colonies growing on appropriate minimal medium containing (+ura) or not (−ura) uracil. Strain carrying pEE-URA3 was used as a control. Strain carrying pEgpIIE-URA3 splicing reporter was transformed or not with the NLS-IEP^co^ expressing plasmid (pNLS-IEP^co^). Three resulting transformants were cultured until OD_600 nm_ reached 2.5 and applied on the appropriate minimal medium using a 10^−4^ dilution of the culture. Data indicate the numbers of yeast colonies on the plate per ml of the dilution.

To study the expression and splicing of the Pl.LSU/2 intron in yeast, we developed a *URA3*-based intron-splicing reporter expressed from a 2-µ plasmid (Fig. 4A; pEgpIIE-URA3; Table S1 A). The Pl.LSU/2 (DIVab) intron flanked by its natural exons (50 last nucleotides of exon 2 and 71 first nucleotides of exon 3) [54] was fused to the coding sequence of URA3, which encodes a orotidine 5-phosphate decarboxylase (Ura3p). The *URA3* gene is read in-frame only upon precise Pl.LSU/2 splicing, which should lead to Ura3p expression and growth of an ura3- (*ura3*Δ*0*) strain of *S. cerevisiae* on minimal media lacking uracil (Fig. 4A). Expression of the Ura3p can be detected with an HA tag added upstream of the exon 2, generating the fusion HA-E2-E3-Ura3p. A control plasmid (pEE-URA3) was also used in this assay to ensure that the hybrid HA-E2-E3-URA3 protein is able to complement the ura3- mutation.

Previous studies have shown that optimal splicing of the bacterial Ll.LtrB group II intron in bacteria and yeast requires the maturase activity of its encoded protein, LtrA, by promoting the folding of the intron RNA into its catalytically active structure. The open reading frame of Pl.LSU/2 intron contains a conserved X domain similar to that of LtrA. Therefore, to determine if Pl.LSU/2 IEP has maturase activity facilitating the splicing of its intron, we first attempted to demonstrate intron splicing through functional restoration of yeast growth in the *URA3*-based intron-splicing reporter assay. In this system, the IEP protein was conditionally expressed using an inducible GAL10 promoter plasmid (Fig. 4B; pNLS-IEP^co^) and intron sequences were delivered *in trans*. For these experiments, we used an IEP sequence that was codon-optimized for translation in human cells (IEP^co^) tagged in C-terminus with c-Myc epitopes, and containing nuclear localization signals (NLS) of the SV-40 T-antigen (Fig. 4B) to address the protein to the nucleus. The inducible/repressible expression of the NLS-IEP^co^ was confirmed respectively in galactose (Gal) and glucose (Glc) media and nuclear localization of the protein was verified by western blot analysis on yeast nuclear protein extracts (data not shown).

Yeasts were thus transformed with either the control pEE-URA3 plasmid or the pEgpIIE-URA3 plasmid. Subsequently, the NLS-IEP^co^ expressing plasmid was transformed or not in yeast carrying pEgpIIE-URA3 and the number of colonies on medium containing or not uracil was determined for each condition (Fig. 4C). Functional restoration of growth could not be demonstrated in the *URA3*-based intron splicing reporter assay. Yeasts expressing the EgpIIE-URA3 cassette repeatedly fail to grow on the –ura selective medium, even when EgpIIE-URA3 is coexpressed with NLS-IEP^co^ (Fig. 4C). However, yeasts grow on minimal medium lacking uracil when the cells are transformed with the pEE-URA3 plasmid encoding the hybrid E2-E3-URA3 protein proving the orotidine 5-phosphate decarboxylase activity of the hybrid protein, as it confers the ability to complement the ura- mutation (Fig. 4C). Thus, the splicing of the Pl.LSU/2 intron could not be demonstrated through this assay into yeast cells.

### Pl.LSU/2 Group II Intron can Splice in Yeast in an IEP-dependent Manner

**Figure 5:**
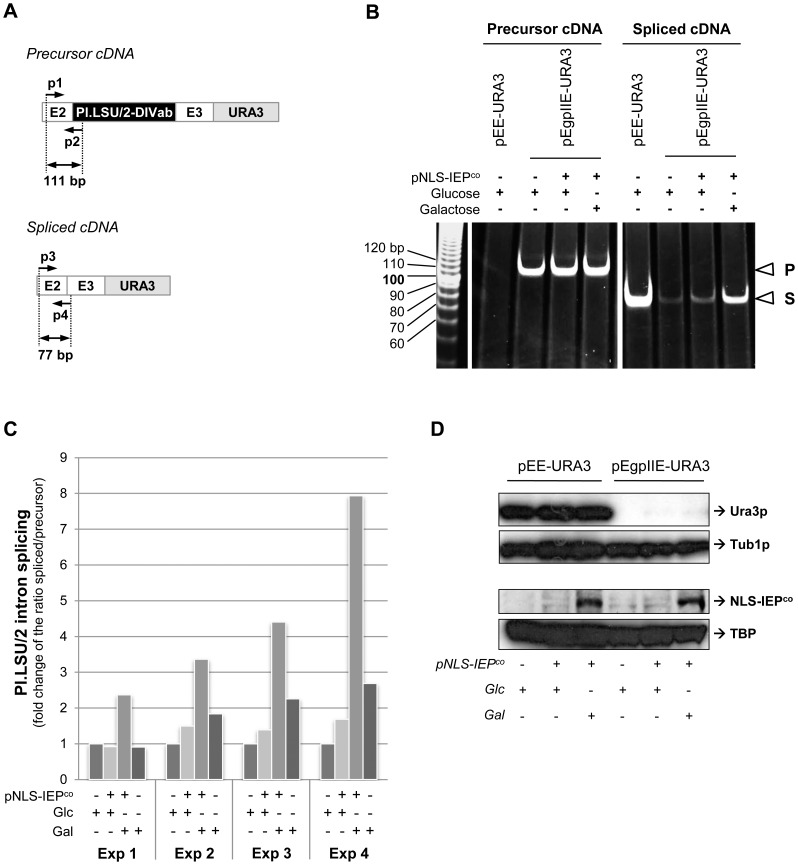
IEP-mediated Pl.LSU/2 splicing *in vivo*. (*A*) Schematic representation of amplification products from precursor and spliced cDNA obtained by RT-PCR. Black rectangle: Pl.LSU/2 intron-DIVab; E2 and E3∶50 last nt of exon 2 and 71 first nt of exon 3; gray rectangle: URA3 ORF; black arrow: primer; p1 and p2: forward and reverse primers amplifying cDNA derived from the precursor mRNA (111 bp product); p3 and p4: forward and reverse primers amplifying cDNA derived from the spliced mRNA (77 bp product). (*B*) Acrylamide electrophoresis of RT-PCR products.Total RNA were extracted from yeast carrying pEE-URA3 or pEgpIIE-URA3 and transformed (+) or not (−) with the NLS-IEP^co^ expressing plasmid (pNLS-IEP^co^). Cells were grown in presence (+) or absence (−) of glucose or galactose (inducer of the NLS-IEP^co^ expression). P: amplification product from precursor cDNA; S: amplification product from spliced cDNA. Numbers at *left* indicate molecular mass marker in base pair (bp). (*C*) Quantification of the Pl.LSU/2 *in vivo* splicing by RT-qPCR. cDNA copy number obtained from spliced and precursor mRNA were calculated using standard amplification curves made by serial dilutions of SphI-linearized pEE-URA3 and pEgpIIE-URA3 plasmids, respectively. Ratios of spliced/precursor cDNA copy number were determined and data were normalized on condition 1 (pNLS-IEPco (−); Glc+; Gal−). Four independent experiments are represented (Exp 1 to Exp 4). (*D*) Western blot analysis of Ura3p made from pEE-URA3 or pEgpIIE-URA3. Yeast strains were cultivated in glucose (Glc+; Gal−) or in galactose (Glc−; Gal+) and in absence (−) or presence (+) of pNLS-IEP^co^. Tubulin 1 protein (Tub1p) expression was also determined, as well as nuclear expression of NLS-IEP^co^ and TATA-Binding protein (TBP).

Since the assay reads-out both RNA splicing and translation of the spliced mRNA, further investigation was conducted to determine if RNAs were transcribed, spliced and translated. To determine if the Pl.LSU/2 intron was spliced from the precursor mRNA, an RT-PCR analysis was performed on RNA extracted from yeast cells harboring either pEE-URA3 or pEgpIIE-URA3 expressed in the presence or absence of NLS-IEP^co^. Two different pairs of primers were used to specifically amplify cDNA obtained from precursor or spliced mRNA (Fig. 5A; Table S1 B). We first show that the pEgpIIE-URA3 splicing reporter allows the expression of precursor mRNA in all conditions (Fig. 5B; precursor cDNA, pEgpIIE-URA3). As expected, the control plasmid pEE-URA3 allows the expression only of a mRNA corresponding to the spliced mRNA (Fig. 5B; pEE-URA3). In absence of NLS-IEP^co^, a poorly efficient splicing of Pl.LSU/2 can be detected from yeast harbouring the pEgpIIE-URA3 reporter (Fig. 5B; spliced cDNA, pEgpIIE-URA3). It is worth noting that splicing efficiency is significantly increased when NLS-IEP^co^ is expressed into yeast cells (Fig. 5B; spliced cDNA, Glucose−/Galactose+). Accurate Pl.LSU/2 splicing was verified by sequencing across the splice junction of the RT-PCR spliced products (data not shown). These results were quantified using an RT-qPCR analysis performed on four independent experiments (Fig. 5C). Precursor and spliced cDNA copy numbers were calculated and ratios of spliced/precursor cDNA were determined to measure splicing efficiency. We confirm that in absence of NLS-IEP^co^ expression (Glc+/Gal−), the splicing of Pl.LSU/2 is poorly efficient while the Pl.LSU/2 intron splicing efficiency in yeast cells is significantly increased upon NLS-IEP^co^ expression (Fig. 5C; Glc−/Gal+; between 2.1 and 7.9 fold change compared to background; p<0,032, *t*-test using a unilateral pair-wise comparison). These results indicate first that the Pl.LSU/2 intron can be expressed in yeast and also that the IEP presents a maturase activity *in vivo*.

### Spliced Pl.LSU/2 Group II Transcripts are not Translated

The results of the phenotypic analysis showing an absence of clones on medium lacking uracil (Fig. 4C) and the level of splicing observed in yeast harboring the pEgpIIE-URA3 reporter and expressing the NLS-IEP^co^ (Fig. 5C) suggested a blockade in expression of the spliced messenger. Indeed, we had not been able to detect by western blot the Ura3p hybrid protein resulting theoretically from the Pl.LSU/2 spliced mRNA (Fig. 5D; pEgpIIE-URA3, Ura3p), even upon robust NLS-IEP^co^ expression (Fig. 5D). In contrast, high levels of Ura3p hybrid protein are expressed in yeast harboring pEE-URA3 (Fig. 5D). The absence of detectable level of Ura3p in yeast carrying pEgpIIE-URA3 thus explains the failure of functional growth restoration in the *URA3*-based intron splicing reporter assay.

### Splicing Assay of Pl.LSU/2 Intron in Human Cells

**Figure 6:**
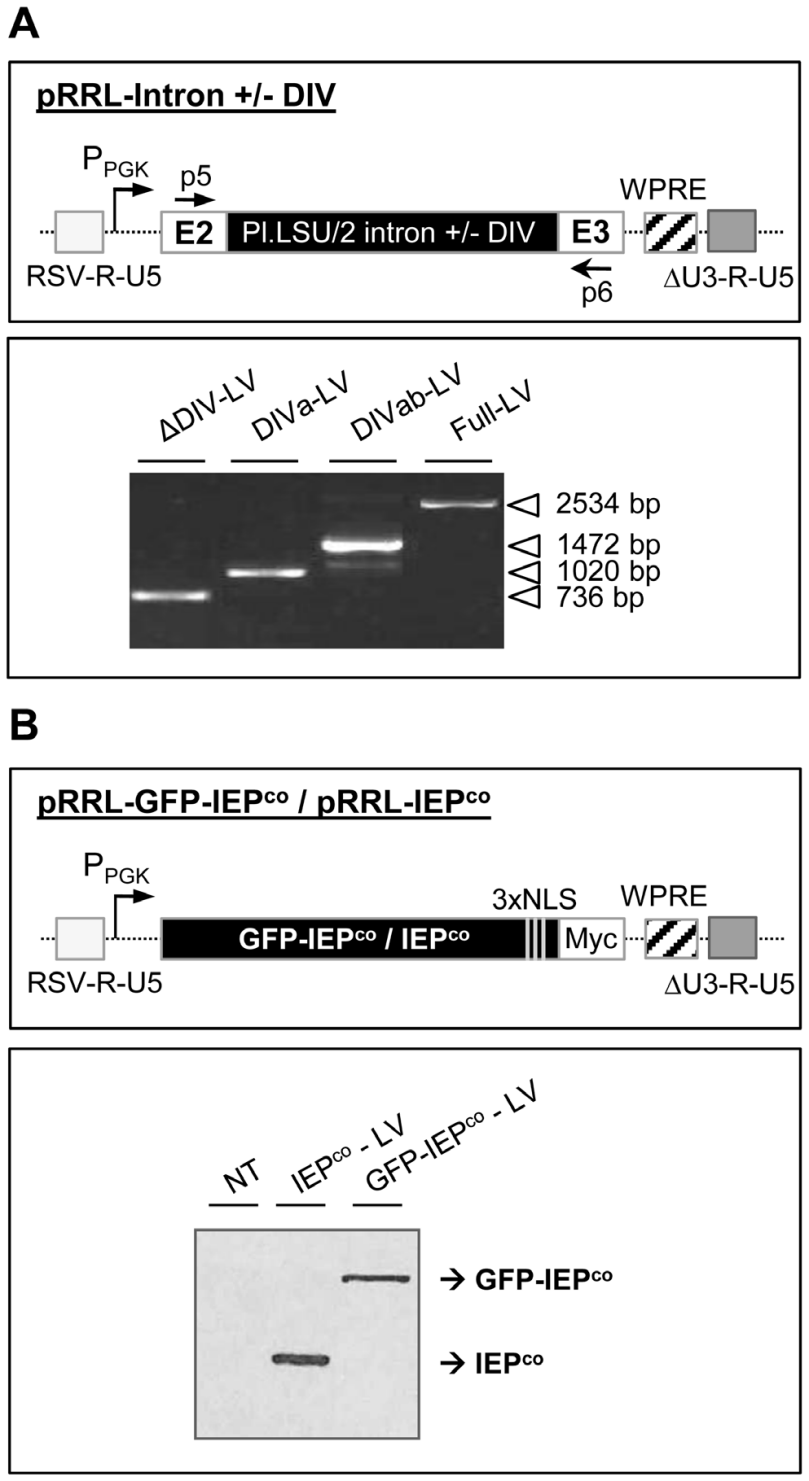
*In vivo* splicing assay of Pl.LSU/2 intron in human cell lines. (*A*) *Upper panel.* Schematic representation of Pl.LSU/2 intron transfer cassettes (pRRL-Intron +/− DIV) used in this study. Several sizes of the intron domain IV are used (See Fig. 1B; ΔDIV, DIVa, DIVab and full length DIV; Table S1 A). HCT 116 cells were transduced by the corresponding VSV-G-pseudotyped lentiviral vectors (LVs) to establish stable cell lines expressing the four different forms of Pl.LSU/2 intron. Light gray rectangle : chimeric 5′ LTR (RSV-R-U5); P_PGK_: phosphoglycerate kinase gene promoter; E2 and E3∶50 last nt of exon 2 and 71 first nt of exon 3; black rectangle : Pl.LSU/2 intron with various size of the DIV (See Fig. 1B); WPRE: Woodchuck hepatitis post-transcriptional regulation element; p5 and p6: forward and reverse primers amplifying cDNA derived from precursor and spliced RNA; dark gray rectangle : 3′ LTR (ΔU3-R-U5). *Lower panel.* Agarose electrophoresis of RT-PCR products. HCT 116 cells were transduced with Pl.LSU/2 intron-expressing LVs (ΔDIV-LV, DIVa-LV, DIVab-LV or Full-LV) and stable cell lines were established. Total RNAs were extracted from the four stable cell lines expressing the different forms of the intron and precursor mRNAs were detected by RT-PCR. (*B*) *Upper panel.* Schematic representation of Pl.LSU/2 IEP gene transfer cassettes used in this study. Black rectangle: codon-optimized sequence of Pl.LSU/2 IEP for translation in human cells, in frame with GFP encoding sequence (GFP-IEP^co^) or not (IEP^co^); 3xNLS: nuclear localization signals; Myc: stretch of 3 c-Myc epitopes. *Lower panel.* Western blot analysis. HCT 116 cell line was either untransduced (NT) or transduced with either IEP^co^-LV or GFP-IEP^co^-LV. Total proteins were extracted from lysates 48 h after transduction and a monoclonal mouse anti-c-myc antibody was used to detect the IEP^co^ and the GFP-IEP^co^.

To study if the splicing of the Pl.LSU/2 intron could also occur in human cells, we first aimed to determine if the Pl.LSU/2 intron could be expressed in the colon carcinoma HCT 116 cell line. We tested various forms of the intron in which the domain IV was more or less extensively deleted (See Fig. 1B; ΔDIV, DIVa, DIVab and full length intron). To stably express the various intron sequences in human cells, we transduced HCT 116 cells with lentiviral gene transfer vectors (LVs) expressing the different forms of the PI.LSU/2 intron flanked by the last 50 nucleotides of exon 2 and the first 71 nucleotides of exon 3 (Fig. 6A; upper panel; pRRL-intron +/−DIV). Transduced HCT 116 cells were expanded for at least ten days to establish stable cell lines expressing the different forms of the intron. RT-PCR using primers p5 and p6 hybridizing E2 and E3, respectively (Fig. 6A; upper panel; Table S1 B) shows expression of the appropriate precursor transcripts in each stable cell lines (Fig. 6A; lower panel; amplicons of 736 bp for ΔDIV intron, 1020 bp for DIVa intron, 1472 bp for DIVab intron and 2534 bp for full length intron). Nevertheless, we did not find any trace of spliced mRNA in any of the stable cell lines analyzed (data not shown). These results suggest that Pl.LSU/2 intron RNA can be expressed but does not splice in human cells.

To determine if the intron-encoded protein could promote splicing of Pl.LSU/2 intron in human cells, we induced the expression of Pl.LSU/2 IEP^co^ or GFP-IEP^co^ fusion protein in human HCT 116 cells by transduction with protein-expressing LVs (Fig. 6B; upper panel). Forty eight hours following transduction of HCT 116 cells with the LV, western blot analysis demonstrates expression of the IEP^co^ or GFP-IEP^co^ in the cells (Fig. 6B; lower panel). GFP expression was also confirmed by western blot using an anti-GFP antibody and by evidence of nuclear localization following microscopy analysis (data not shown).

The previously described Pl.LSU/2 intron expressing stable lines were then transduced by these LVs. Western blot analysis showed the expression of IEP^co^ and GFP-IEP^co^ in these cells (data not shown). To determine the Pl.LSU/2 intron splicing capacity in the presence of IEP, RNAs were extracted and analyzed by RT-qPCR. Primers p7 and p4 (Table S1 B), hybridizing E2 and E2–E3 junction respectively, were used to detect the spliced mRNA. Primers p1 and p2 (Table S1 B), hybridizing E2 and E2-Intron junction respectively, were used to detect the precursor mRNA. Ratios of spliced/precursor cDNA copy number, which determine the splicing efficiency, are less than 2.10^−4^ in every condition tested and for every form of the Pl.LSU/2 intron tested (data not shown). This result shows that, in contrast to yeast, the Pl.LSU/2 intron is unable to splice efficiently in human cells in this context, even in presence of the intron-encoded protein that was codon-optimized for translation in human cells.

## Discussion

This study provides the first functional description of the ribozyme activity of the Pl.LSU/2 group II intron *in vivo*. A catalytically-active recombinant PI.LSU/2 intron-encoded protein can be produced in *E. coli* and purified. This protein presents a reverse transcriptase activity *in vitro* both alone or when coexpressed with the intronic RNA. It also displays a maturase activity which facilitates the splicing of the Pl.LSU/2 intron *in vivo* in yeast in a IEP dose-dependent manner.

These results contribute to characterize the poorly studied Pl.LSU/2 intron. Prior to our study, no information was yet available on the *in vivo* properties of this intron, neither in its natural environment nor in experimental systems. The description of the RT and maturase activities of the IEP confirms some of the predicted properties encoded by the domain IV of the intron which was known to contain an open reading frame theoretically encoding a protein presenting the four characteristic domains (RT, X/maturase, D and En) of other group II intron-encoded proteins [55], [56].

One limitation of the use of group II introns in eukaryotes could be the requirement for a correct folding of both the IEP and the intron RNA, therefore it is important to assess the cooperation between the intron and IEP. Here, we show for the first time that the Pl.LSU/2 IEP displays a RT activity when complexed as RNP. In addition, the PI.LSU/2 IEP has an intrinsic RT activity *in vitro* in the absence of the intron RNA. However, one can postulate that the presence of nucleic acids in the sucrose cushion purification fractions may participate in the Pl.LSU/2 IEP stabilization and activity. In previous studies of the Lambowitz group [23], [53], [65], the *Lactococcus lactis* Ll.LtrB IEP (LtrA) correct folding was demonstrated by its RT activity either alone or when coexpressed with its intron RNA. Moreover, highly active LtrA was recently obtained after nucleic acids removal prior to purification [65], showing that this protein does not require bounded nucleic acids to be stabilized. Similarly, Vellore et al. expressed in *E. coli* the G.st.I1 intron-encoded protein (called *trt*) from *Geobacillus stearothermophilus* fused to a 6xHis tag without co-expressing the intron RNA [68]. The authors showed an RT activity using partially purified protein fraction. In the case of Pl.LSU/2, the IEP reverse transcriptase activity could be clearly demonstrated in the absence of intronic RNA, showing the PI.LSU2 IEP self-activation properties.

The biochemical properties of this IEP may also facilitate its use *in vivo*. The PI.LSU/2 intron can be engineered to splice *in vivo* in yeast and this is facilitated in a dose-dependent manner by the Pl.LSU/2 IEP. Although the Pl.LSU/2 IEP encoding gene used here is a codon-optimized sequence for translation in human cells, IEP translation in yeast is enough efficient to improve splicing of Pl.LSU/2. Presumably the maturase activity of the PI.LSU/2 IEP promotes the folding of the intron RNA into its catalytic tertiary structure *in vivo.* Interestingly, a residual splicing could be detected by RT-PCR and RT-qPCR using RNA extracts from yeasts that do not express the IEP. This IEP-independent splicing could have occur *in vivo* (which would be consistent with the ability of this intron to splice *in vitro* even under not optimal Mg^2+^ conditions [54]) or *in vitro* during the RT-PCR reactions precisely because this ribozyme is highly active *in vitro*.

In spite of evidence of functional properties of the PI.LSU/2 intron, we failed to develop a productive system for genomic targeting at this stage. While we demonstrated the presence of a spliced mRNA in yeast cells, there was no translation of the spliced mRNA since restoration of the URA3 ORF did not lead to the expected growth on a minimal medium without uracil. It is possible that the yield of spliced mRNA in yeast cells is not sufficient to the expression of a detectable amount of Ura3p proteins by western blot and that the yield of expressed Ura3p does not reach the required threshold for yeast growth on medium lacking uracil. However, the possibility of a translation blockade is reminiscent of results already reported by Chalamcharla et al. with the Ll.LtrB intron using different reporter genes [69]. The authors demonstrated that Ll.LtrB intron splicing occured predominantly in the cytoplasm of yeast cells and that precursor mRNA was subjected to nonsense-mediated mRNA decay (NMD). They also showed that the spliced mRNA was subjected to an NMD-independent translation blockade. However, the mechanism involved remains unknown. The authors speculated that the pairing mechanism between the intron lariat and the spliced mRNA via EBS (exon binding sites)/IBS (intron binding sites) interactions [6] could impede the spliced mRNA translation [69]. One would also postulate that the translation blockade could result from RNA sequestration to cytoplasmic microdomains such as P-bodies or stress granules that play important role in mRNA processing, including repression of translation and mRNA decay [70]–[72]. Anyway, the fact that this translation blockade also occurs through the use of Pl.LSU/2 group II intron supports the Chalamcharla et al. hypothesis that the mechanism involved is transposable to other group II introns and may has impeded their spread in eukaryotic nuclear genes.

We then tested the ability of the intron to splice in human cells. Here, we used different forms of the Pl.LSU/2 intron with deletions of various sizes of the domain IV in order to determine if one of these parts are required for intron high-affinity binding to the IEP. The domain IV of the Ll.LtrB intron is known to bind its IEP [73], but is not needed for *in vitro* splicing, as in absence of it, a residual splicing occurs [27], [67]. In the same way, the domain IV of the yeast *coxI*-I2 intron is required for stable binding of its IEP and additional contacts with the catalytic core of the intron promote the splicing [74]. The domain IV of group II introns appears to guide the interactions or anchor the IEP to catalytic core regions of the intron. In spite of the use of various domain IV coding regions, in spite of using a humanized codon-optimized IEP, in spite of evidence of expression of the IEP and intron in human cells and in spite of the expected nuclear localization of the recombinant IEP in human cells, we failed to detect any splicing of Pl.LSU/2 intron in human cells. Misfolding of IEP and/or intron RNA could explain this splicing defect, but alternatively, defective nuclear/cytoplasmic compartimentalization or inadapted environnement could also be implicated.

The adaptation of group II introns homing mechanism could be considered in the context of a gene repair strategy approach in gene therapy. In current gene therapy assays, the transgene integration with retroviral vectors is not site-specific. Expression of therapeutic transgenes is sometimes unregulated due to *cis*-acting elements present in the neighbouring of the insertion site. Insertions near oncogenes also present a risk of activation by promoters or enhancers carried by the vector [75]–[80]. All of these issues could be circumvented by targeting the insertion into the original site (gene repairing). The repaired wild type copy will be under the control of original cis-regulating sequences. Today, strategies based on genome double strand breaks (DSB) in order to achieved reparation by homologous recombination are thouroughly investigated. The use of group II intron could be an alternative approache avoiving the putative genotoxicity due to off-target DSB.

We present encouraging data that suggest that PI.LSU/2 group II intron could have advantageaous qualities for engineering genomic targeting strategies. This intron and its IEP function *in vivo* in yeast. However, the use of Pl.LSU/2 and other group II introns in human genomic engineering will require further optimizations.

## Supporting Information

Figure S1 **Nucleic acids in IEP and RNPs purified fractions.** Agarose gel electrophoresis of 1 µg and 250 ng of nucleic acids in IEP and RNPs purified fractions (WT and mtDD-) respectively, obtained after ultracentrifugation in sucrose cushion. Numbers at *left* indicate size of DNA ladder in base pair (bp).
(TIF)

Figure S2 **Secondary structure of the Pl.LSU/2 intron domain IV predicted by sFold.** Pl.LSU/2 intron domain IV (DIV; 1870 nts) predicted RNA secondary structure obtained with the sFold software (http://sfold.wadsworth.org). The domain IV used is from nucleotide 494 to nucleotide 2363 of the Pl.LSU/2 intron.
(TIF)

Table S1 Plasmid and oligonucleotids used in this work. (*A*) Relevant characteristics of plasmids used in this work. References cited in the last column are listed at the end of the table. (*B*) DNA sequence of oligonucleotides used in this work.
(DOCX)
