# Supplementary figures and images for: The Brown Algae Pl.LSU/2 Group II Intron-Encoded Protein Has Functional Reverse Transcriptase and Maturase Activities
Source: PLoS One. 2013 Mar 11;8(3):e58263. doi: 10.1371/journal.pone.0058263 (PMC3594303; doi:10.1371/journal.pone.0058263)

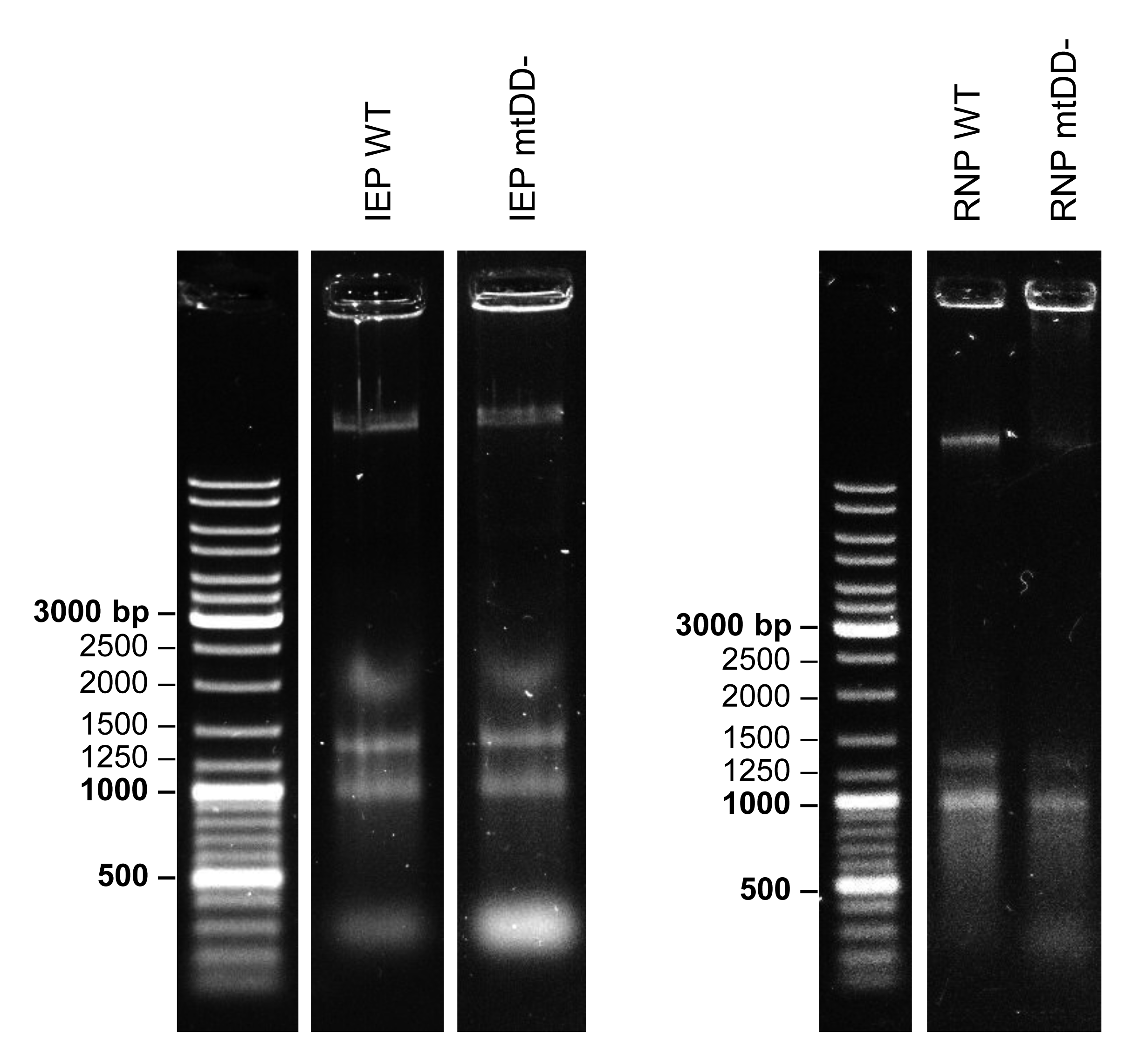

Supplement: Figure S1 — Nucleic acids in IEP and RNPs purified fractions. Agarose gel electrophoresis of 1 µg and 250 ng of nucleic acids in IEP and RNPs purified fractions (WT and mtDD-) respectively, obtained after ultracentrifugation in sucrose cushion. Numbers at left indicate size of DNA ladder in base pair (bp). (TIF) [file pone.0058263.s001.tif]

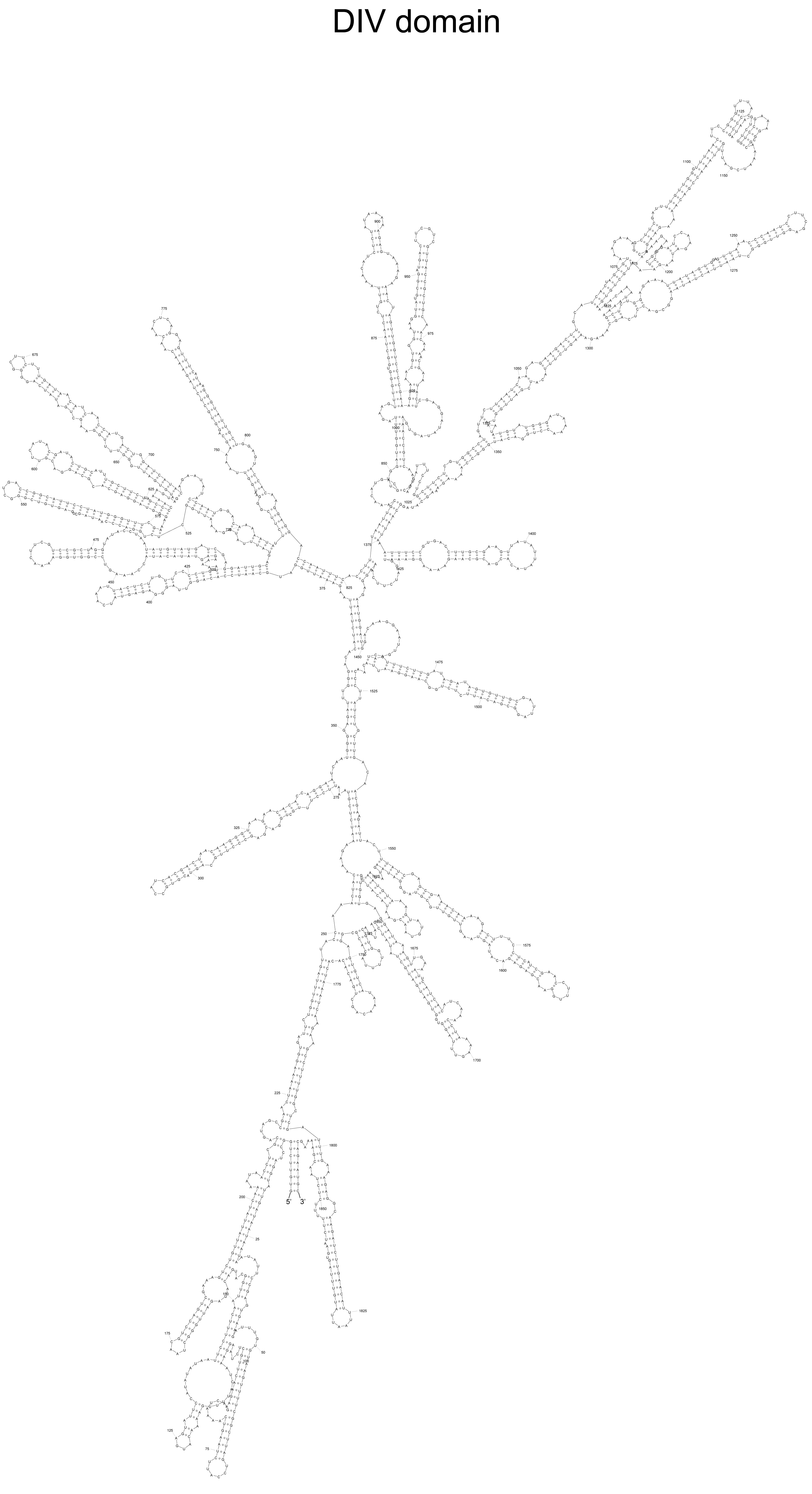

Supplement: Figure S2 — Secondary structure of the Pl.LSU/2 intron domain IV predicted by sFold. Pl.LSU/2 intron domain IV (DIV; 1870 nts) predicted RNA secondary structure obtained with the sFold software (http://sfold.wadsworth.org). The domain IV used is from nucleotide 494 to nucleotide 2363 of the Pl.LSU/2 intron. (TIF) [file pone.0058263.s002.tif]
