# Supplementary material for: The Brown Algae Pl.LSU/2 Group II Intron-Encoded Protein Has Functional Reverse Transcriptase and Maturase Activities
Source: PLoS One. 2013 Mar 11;8(3):e58263. doi: 10.1371/journal.pone.0058263 (PMC3594303; doi:10.1371/journal.pone.0058263)
Supplement: Table S1 — Plasmid and oligonucleotids used in this work. (A) Relevant characteristics of plasmids used in this work. References cited in the last column are listed at the end of the table. (B) DNA sequence of oligonucleotides used in this work. (DOCX) [file pone.0058263.s003.docx]

**Supplemental Table S1. Plasmids and oligonucleotides used in this work**

**A. Plasmids**

| **Plasmid** | **Relevant characteristics** | **Reference** |
| --- | --- | --- |
| pET151/D-TOPO | *E. coli* T7 expression vector allowing a directional TOPO cloning in frame of a 6xHis/V5 tag. | Invitrogen |
| p151-IEP | Pl.LSU/2 IEP fused to a 6xHis/V5 tag in its N-terminus and expressed from a T7 promoter on the pET151 plasmid. | This work |
| p151-IEPmtDD- | p151-IEP derivative vector in which the conserved YADD motif of the IEP RT domain is changed in YAAA. The resulting IEP mtDD- protein should be RT-defective. | This work |
| p151-E+I+IEP | Pl.LSU/2 full length group II intron expressed from a T7 promoter on the pET151 plasmid and containing the IEP fused to a 6xHis/V5 tag in its N-terminus. | This work |
| p151-E+I+IEPmtDD- | p151-E+I+IEP derivative in which the conserved YADD motif of the IEP RT domain is changed in YAAA. | This work |
| pBFG1 | 2µ plasmid containing the LEU2 gene selection marker, 3 HA epitopes and a PGK promoter. | ([Yelin et al. 1999](#_ENREF_4)) |
| pCI-neo | Mammalian expression vector containing a neomycin phosphotransferase gene. | Promega |
| pCIneo-E2E3mGFP | In frame Pl.LSU/2 flanking exons E2 (the last 50 nt) and E3 (the first 71 nt) containing a mutation that converts a STOP codon in E3 in tyrosine (E3m) and fused to the 5’-end of the GFP ORF on the pCI-neo plasmid. | This work |
| pCIneo-E2IntronDIVabE3mGFP | pCIneo derived plasmid containing a Pl.LSU/2 intron deleted form in which section from nucleotide 369 to nucleotide 1446 of DIV domain was removed (intron DIVab), flanked by the last 50 nt of E2 and the first 71 nt of E3 with a mutation that converts a STOP codon in tyrosine (E3m) and fused to the 5’-end of the GFP ORF. | This work |
| pEE-URA3 | Pl.LSU/2 flanking exons (the last 50 nt of E2 and the first 71 nt of E3m) fused to 3 HA epitopes in its N-terminus and to a URA3 gene lacking the start codon in its C-terminus expressed from a PGK promoter on the pBFG1 plasmid. | This work |
| pEgpIIE-URA3 | URA3-based PL.LSU/2 intron splicing reporter. The last 50 nt of E2, the Pl.LSU/2 intron DIVab form, see above) and the first 71 nt of E3 with a mutation that converts a STOP codon in tyrosine (E3m) are cloned just downstream of a URA3 gene lacking the start codon. The whole cassette is expressed from a PGK promoter on the pBFG1 plasmid. | This work |
| pNLS-IEP^co^ | Codon-optimized Pl.LSU/2 NLS-IEP fused to a c-myc epitope in its C-terminus and expressed from the GAL10 promoter of the pYEF1 plasmid on the pRS413 plasmid. | This work |
| pPl.LSU/2 | Cloning plasmid containing the Pl.LSU/2 group II intron flanked by the last 50 nt of exon 2 and the first 71 nt of exon 3. | (Costa et al. 1997) |
| pPl.LSU/2-∆DIV | pPl.LSU/2 derivative construct in which most of the intron DIV domain was removed : section from nucleotide 8 to nucleotide 1818 of the DIV domain was replaced by CCTAGGATCT. | (Costa et al. 1997) |
| pRRL-backbone | Advanced generation SIN Tat-independent HIV lentiviral vector system. | (Charrier et al. 2007) |
| pRRL-intron-∆DIV | pRRL-backbone derived plasmid containing containing the Pl.LSU/2 group II intron with ∆DIV deletion (see above) flanked by the last 50 nt of exon 2 and the first 71 nt of exon 3. | This work |
| pRRL-intron-DIVa | pRRL-backbone derived plasmid containing the last 50 nt of exon 2, a Pl.LSU/2 intron deleted form in which section from nucleotide 244 to nucleotide 1772 of DIV domain was removed, and the first 71 nt of exon 3. | This work |
| pRRL-intron-DIVab | pRRL-backbone derived plasmid containing the last 50 nt of exon 2, the Pl.LSU/2 intron DIVab form (See above) and the first 71 nt of exon 3. | This work |
| pRRL-intron-Full | pRRL-backbone derived plasmid containing Pl.LSU/2 group II intron flanked by the last 50 nt of exon 2 and the first 71 nt of exon 3. | This work |
| pRRL-GFP | pRRL-backbone derived plasmid containing GFP ORF | This work |
| pRRL-GFP-IEP^co^ | pRRL-backbone derived plasmid containing codon-optimized Pl.LSU/2 IEP ORF fused to the GFP ORF in its N-terminus and to3 NLS and a c-myc epitope in its C-terminus. | This work |
| pRRL-IEP^co^ | pRRL-backbone derived plasmid containing codon-optimized Pl.LSU/2 IEP fused to 3 NLS and a c-myc epitope in its C-terminus. | This work |
| pRS413 | ARS-CEN plasmid with the HIS3 gene selection marker. | ([Sikorski and Hieter 1989](#_ENREF_3); [Christianson et al. 1992](#_ENREF_1)) |
| pRS426 | 2µ plasmid containing the URA3 gene selection marker. | ([Christianson et al. 1992](#_ENREF_1)) |
| pUC57 | *E. coli* cloning plasmid. | Genescript |
| pUC57-NLS-IEP^co^ | Codon-optimized Pl.LSU/2 IEP with 3 NLS and a c-myc epitope in its C-terminus. | This work |
| pYEF1 | 2µ plasmid containing the GAL10 promoter and terminator. | ([Cullin and Minvielle-Sebastia 1994](#_ENREF_2)) |

**B. Oligonucleotides**

| **Oligonucleotide** | **Sequence (5’ to 3’)** | **Use** |
| --- | --- | --- |
| BamHI-K-IEP | GGGATCCACCATGAGTATTCCTTACATAATTCCG | Amplification of IEP^co^ ORF |
| DM2 | GTAGCTTTCGAAGCTTTACCTGCCGGCACC | Amplification of E2E3m and E2-IntronDIVab-E3m |
| DM3 | GGTAAAGCTTCGAAAGCTACATATAAGGAA | Amplification of URA3 ORF |
| DM4 | GAATTCAGTTTTTTAGTTTTGCTGG | Amplification of URA3 ORF |
| EcoRI-Stop-Myc | AGAATTCCTAGGCAGCGCCGTTCAG | Amplification of IEP^co^ ORF |
| p1 | CTTTTATCTTTGACACAAAATCGGGGG | Amplification of the precursor cDNA (qPCR) |
| p2 | TCCTGAACTTCTTGTCGCACTTTTTA | Amplification of the precursor cDNA (qPCR) |
| p3 | AGGATCCCAGCTTTTATCTTTGACACA | Amplification of E2E3m, E2-IntronDIVab-E3m, and the spliced cDNA (qPCR) |
| p4 | CGAGTTAGCAGAGACCTGTGTTTTTA | Amplification of the spliced cDNA (qPCR) |
| p5 | CTTTTATCTTTGACACAAAATCG | Amplification of the precursor cDNA (PCR) |
| p6 | GCAGGTGTCAGTCCCTATACA | Amplification of the precursor cDNA (PCR) |
| p7 | ATTCACGCGTGGTACCTCTAGAA | Amplification of the spliced cDNA (qPCR) |
| PlLSU2-AS2 | TTAAATGTTCAAGATCTTGC | Amplification of IEP ORF |
| PlLSU2-S2 | CACCATGAGTATTCCATATATA | Amplification of IEP ORF |
| PlLSU2-SacI-AS | CGAGCTCTCGATAAGCTTTACCTGCCG | Amplification of Intron (domains IVb, V and VI)-E3 |
| PlLSU2-XbaI-AS | GCTCTAGAGTTTTCAAAATGATTTCCTTAGAGCAAG | Amplification of E2-Intron (domains I, II, III and IVa) |
| PlLSU2-XbaI-S | GCTCTAGAACTAGTGGATCCCCCGGGCTGCA | Amplification of E2-Intron (domains I, II, III and IVa) |
| PlLSU2-XhoI-S | AAACGAATACTCGAGGATATAGTGAAACCG | Amplification of Intron (domains IVb, V and VI)-E3 |
| RM-R | GTGTGCATTCGTAATGTCTGCCCATTCT | Reverse transcription of total yeast RNA |
| Sal-GAL-F | GTCGACCTAAACTCACAAATTAGAGCTTC | Amplification of the GAL10 promoter and terminator |
| Xba-GAL-R | TCTAGATGTGAGTTAGCTCACTCATTAG | Amplification of the GAL10 promoter and terminator |
| YAAA_F | TGGTAAGGTATGCGGCTGCCTTCGTCGTTACCGC | Site-directed mutagenesis of the YADD motif in the RT domain of IEP |
| YAAA_R | GCGGTAACGACGAAGGCAGCCGCATACCTTACCA | Site-directed mutagenesis of the YADD motif in the RT domain of IEP |

**Supplemental references**

Charrier S, Dupre L, Scaramuzza S, Jeanson-Leh L, Blundell MP, et al. 2007. Lentiviral vectors targeting WASp expression to hematopoietic cells, efficiently transduce and correct cells from WAS patients. *Gene Ther* **14**: 415-428.

Christianson TW, Sikorski RS, Dante M, Shero JH, Hieter P. 1992. Multifunctional yeast high-copy-number shuttle vectors. *Gene* **110**: 119-122.

Costa M, Fontaine JM, Loiseaux-de Goer S, Michel F. 1997. A group II self-splicing intron from the brown alga Pylaiella littoralis is active at unusually low magnesium concentrations and forms populations of molecules with a uniform conformation. *J Mol Biol* **274**: 353-364.

Cullin C, Minvielle-Sebastia L. 1994. Multipurpose vectors designed for the fast generation of N- or C-terminal epitope-tagged proteins. *Yeast* **10**: 105-112.

Sikorski RS, Hieter P. 1989. A system of shuttle vectors and yeast host strains designed for efficient manipulation of DNA in Saccharomyces cerevisiae. *Genetics* **122**: 19-27.

Yelin R, Rotem D, Schuldiner S. 1999. EmrE, a small Escherichia coli multidrug transporter, protects Saccharomyces cerevisiae from toxins by sequestration in the vacuole. *Journal of bacteriology* **181**: 949-956.
